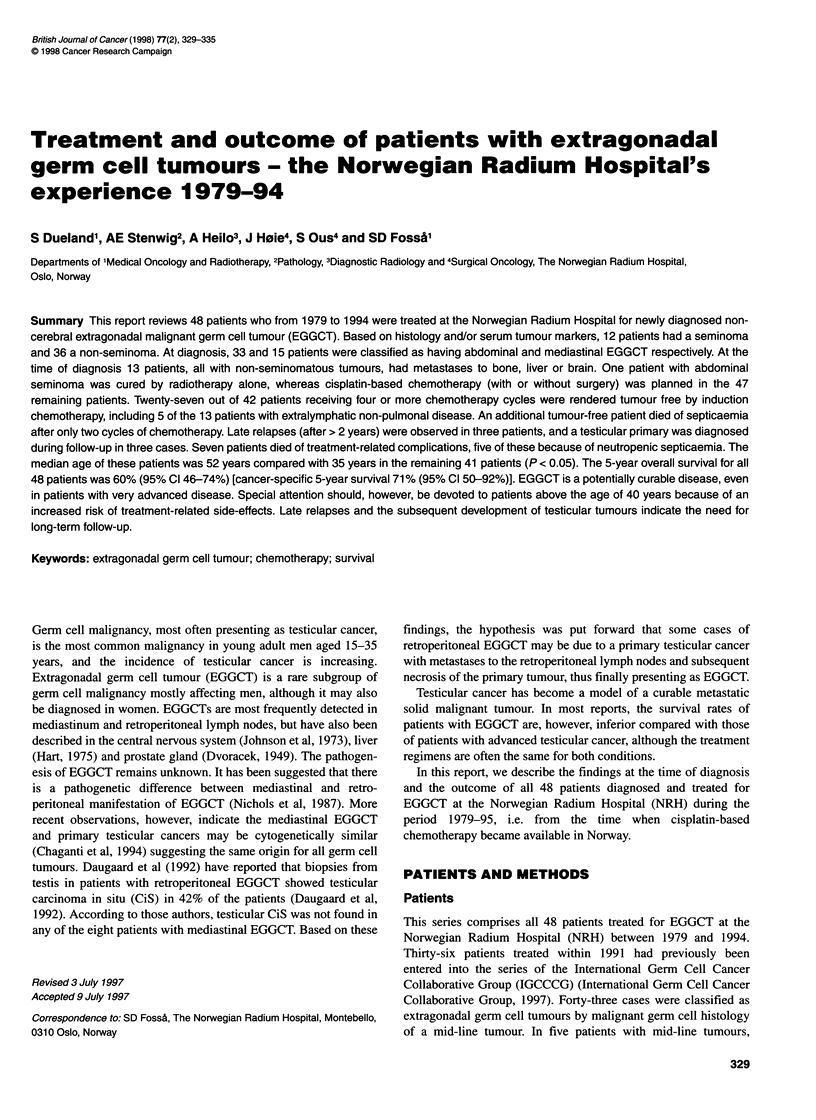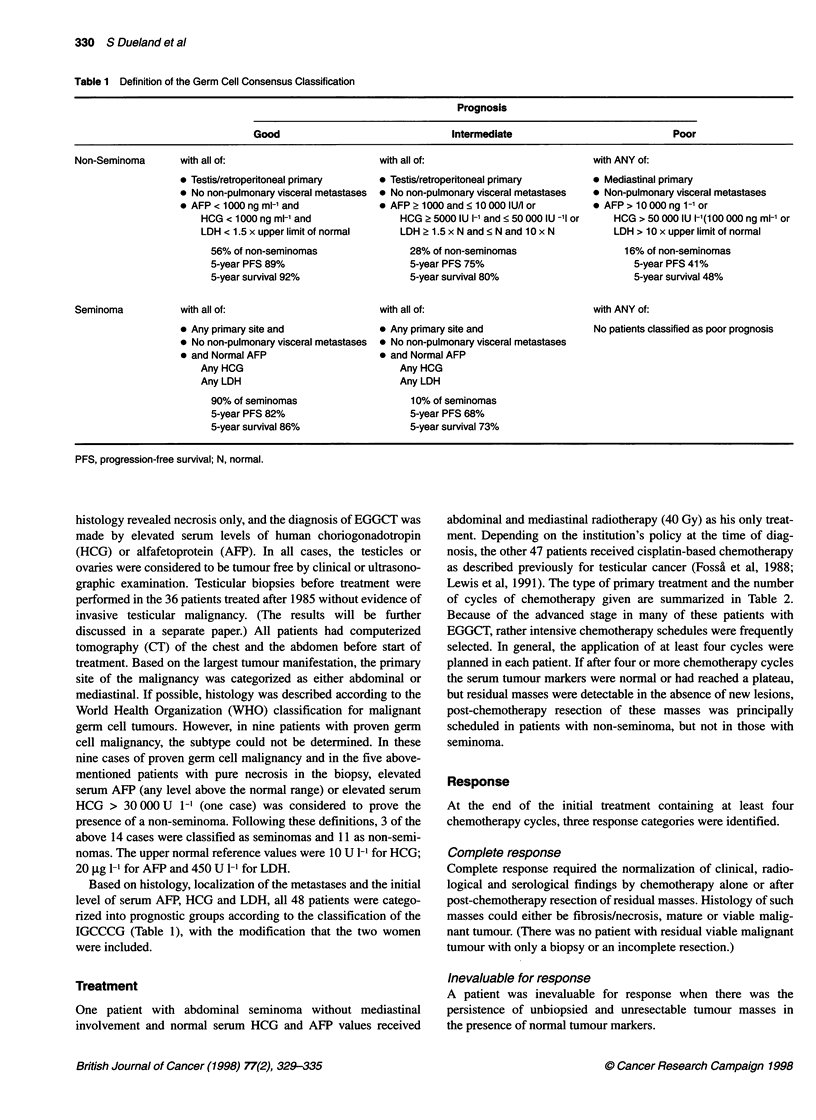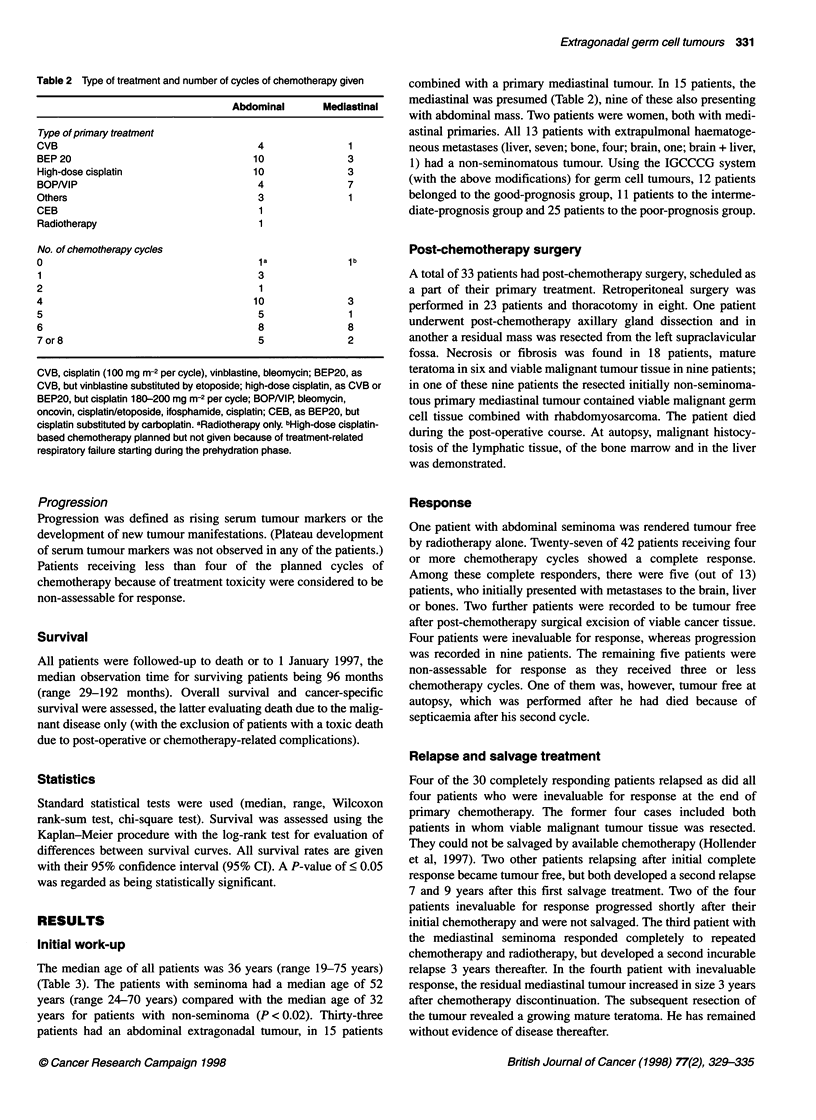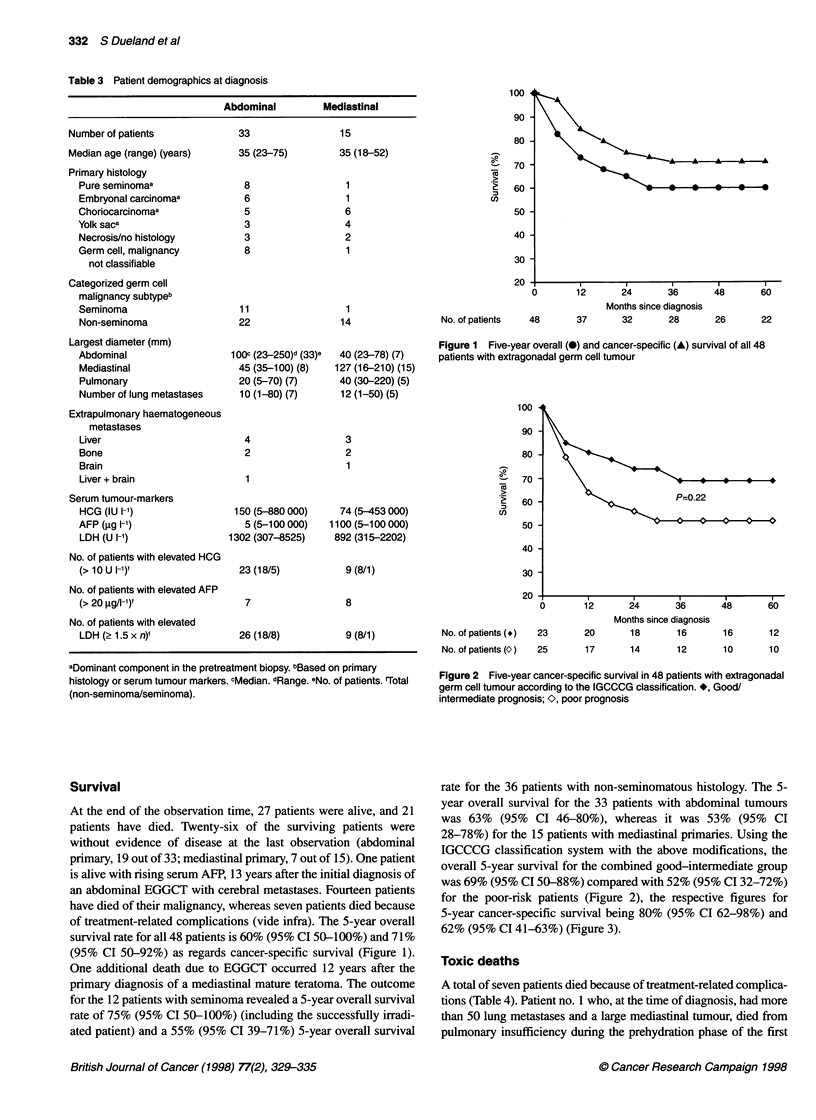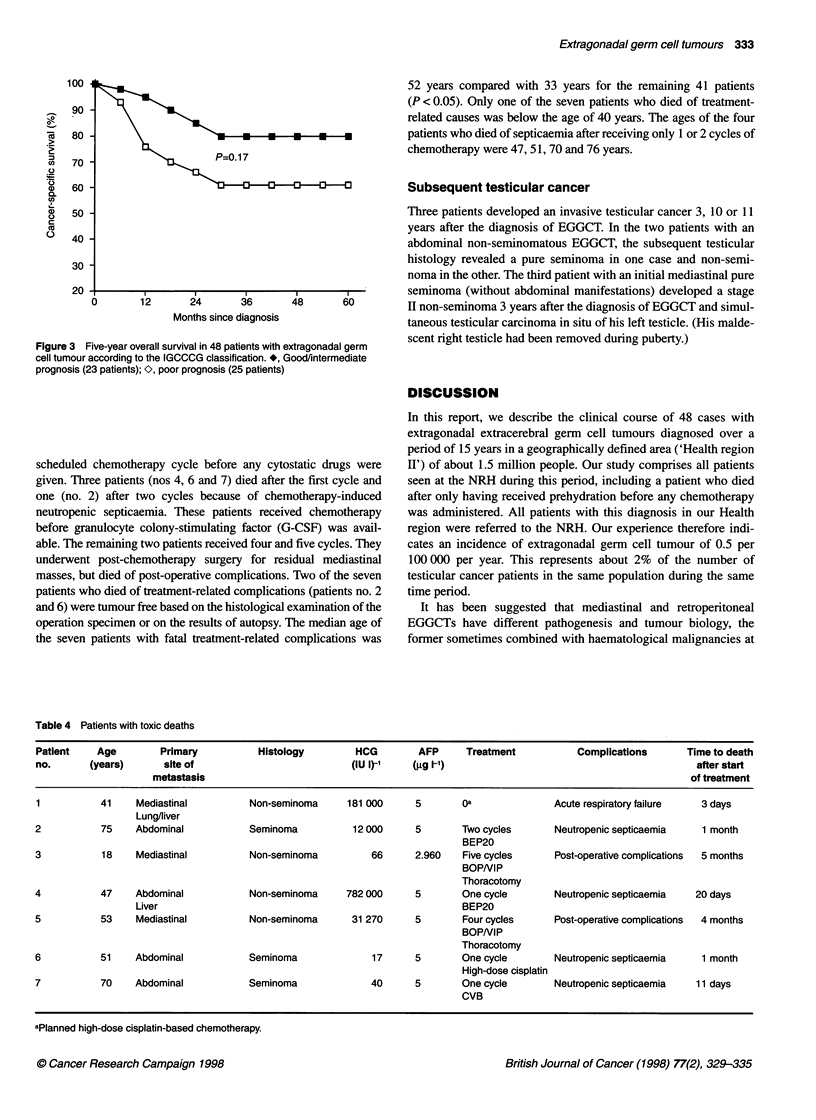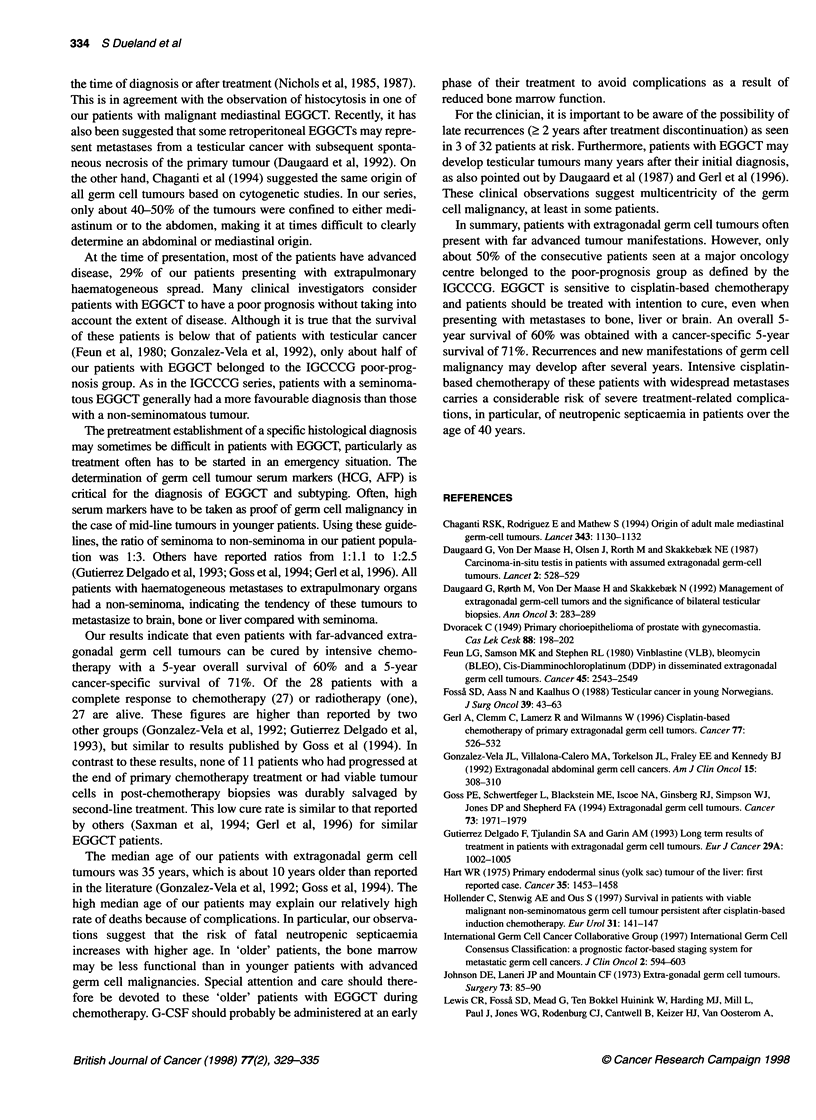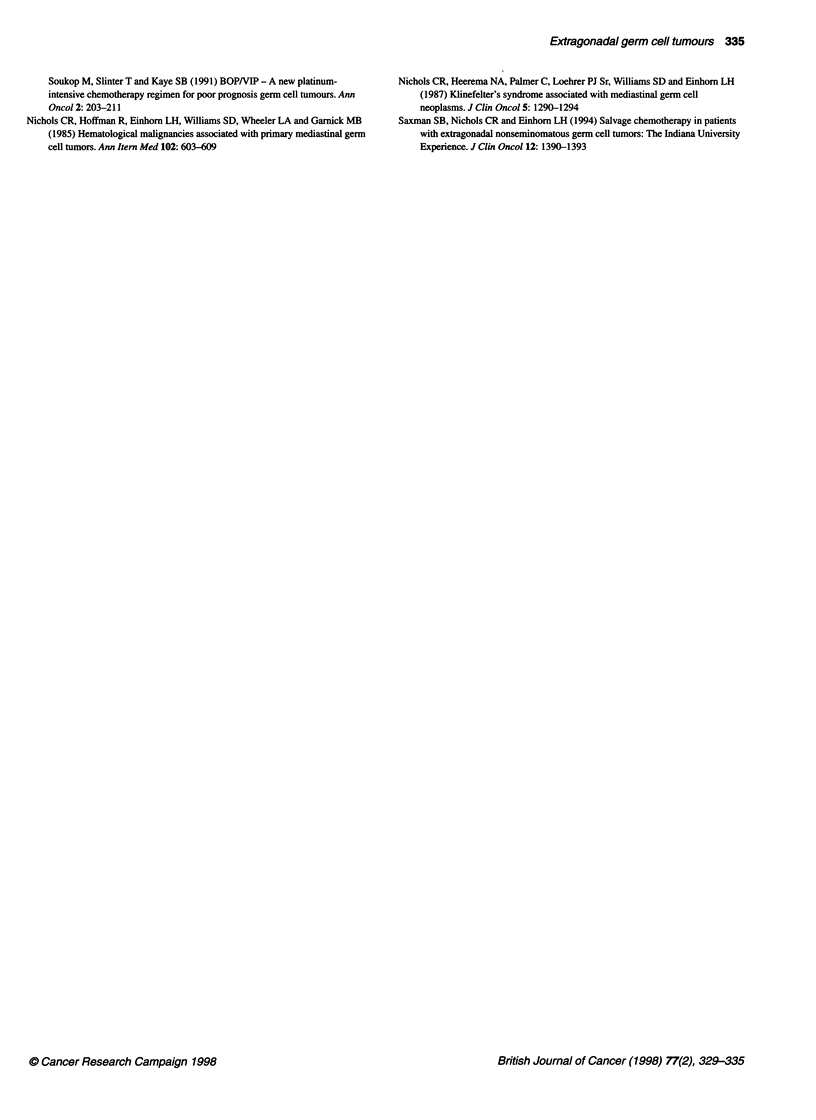# Treatment and outcome of patients with extragonadal germ cell tumours--the Norwegian Radium Hospital's experience 1979-94.

**DOI:** 10.1038/bjc.1998.51

**Published:** 1998

**Authors:** S. Dueland, A. E. Stenwig, A. Heilo, J. HÃ¸ie, S. Ous, S. D. FossÃ¥

**Affiliations:** Department of Medical Oncology, The Norwegian Radium Hospital, Oslo.

## Abstract

This report reviews 48 patients who from 1979 to 1994 were treated at the Norwegian Radium Hospital for newly diagnosed noncerebral extragonadal malignant germ cell tumour (EGGCT). Based on histology and/or serum tumour markers, 12 patients had a seminoma and 36 a non-seminoma. At diagnosis, 33 and 15 patients were classified as having abdominal and mediastinal EGGCT respectively. At the time of diagnosis 13 patients, all with non-seminomatous tumours, had metastases to bone, liver or brain. One patient with abdominal seminoma was cured by radiotherapy alone, whereas cisplatin-based chemotherapy (with or without surgery) was planned in the 47 remaining patients. Twenty-seven out of 42 patients receiving four or more chemotherapy cycles were rendered tumour free by induction chemotherapy, including 5 of the 13 patients with extralymphatic non-pulmonal disease. An additional tumour-free patient died of septicaemia after only two cycles of chemotherapy. Late relapses (after > 2 years) were observed in three patients, and a testicular primary was diagnosed during follow-up in three cases. Seven patients died of treatment-related complications, five of these because of neutropenic septicaemia. The median age of these patients was 52 years compared with 35 years in the remaining 41 patients (P < 0.05). The 5-year overall survival for all 48 patients was 60% (95% CI 46-74%) [cancer-specific 5-year survival 71% (95% CI 50-92%)]. EGGCT is a potentially curable disease, even in patients with very advanced disease. Special attention should, however, be devoted to patients above the age of 40 years because of an increased risk of treatment-related side-effects. Late relapses and the subsequent development of testicular tumours indicate the need for long-term follow-up.


British Journal of Cancer (1998) 77(2), 329–335
© 1998 Cancer Research Campaign

### Treatment and outcome of patients with extragonadal germ cell tumours – the Norwegian Radium Hospital's experience 1979–94

**S Dueland[1], AE Stenwig[2], A Heilo[3], J Høie[4], S Ous[4] and SD Fosså[1]**

Departments of [1]Medical Oncology and Radiotherapy, [2]Pathology, [3]Diagnostic Radiology and [4]Surgical Oncology, The Norwegian Radium Hospital, Oslo, Norway

**Summary** This report reviews 48 patients who from 1979 to 1994 were treated at the Norwegian Radium Hospital for newly diagnosed non-cerebral extragonadal malignant germ cell tumour (EGGCT). Based on histology and/or serum tumour markers, 12 patients had a seminoma and 36 a non-seminoma. At diagnosis, 33 and 15 patients were classified as having abdominal and mediastinal EGGCT respectively. At the time of diagnosis 13 patients, all with non-seminomatous tumours, had metastases to bone, liver or brain. One patient with abdominal seminoma was cured by radiotherapy alone, whereas cisplatin-based chemotherapy (with or without surgery) was planned in the 47 remaining patients. Twenty-seven out of 42 patients receiving four or more chemotherapy cycles were rendered tumour free by induction chemotherapy, including 5 of the 13 patients with extralymphatic non-pulmonal disease. An additional tumour-free patient died of septicaemia after only two cycles of chemotherapy. Late relapses (after > 2 years) were observed in three patients, and a testicular primary was diagnosed during follow-up in three cases. Seven patients died of treatment-related complications, five of these because of neutropenic septicaemia. The median age of these patients was 52 years compared with 35 years in the remaining 41 patients ($P < 0.05$). The 5-year overall survival for all 48 patients was 60% (95% CI 46–74%) [cancer-specific 5-year survival 71% (95% CI 50–92%)]. EGGCT is a potentially curable disease, even in patients with very advanced disease. Special attention should, however, be devoted to patients above the age of 40 years because of an increased risk of treatment-related side-effects. Late relapses and the subsequent development of testicular tumours indicate the need for long-term follow-up.

**Keywords:** extragonadal germ cell tumour; chemotherapy; survival

Germ cell malignancy, most often presenting as testicular cancer, is the most common malignancy in young adult men aged 15–35 years, and the incidence of testicular cancer is increasing. Extragonadal germ cell tumour (EGGCT) is a rare subgroup of germ cell malignancy mostly affecting men, although it may also be diagnosed in women. EGGCTs are most frequently detected in mediastinum and retroperitoneal lymph nodes, but have also been described in the central nervous system (Johnson et al, 1973), liver (Hart, 1975) and prostate gland (Dvoracek, 1949). The pathogenesis of EGGCT remains unknown. It has been suggested that there is a pathogenetic difference between mediastinal and retroperitoneal manifestation of EGGCT (Nichols et al, 1987). More recent observations, however, indicate the mediastinal EGGCT and primary testicular cancers may be cytogenetically similar (Chaganti et al, 1994) suggesting the same origin for all germ cell tumours. Daugaard et al (1992) have reported that biopsies from testis in patients with retroperitoneal EGGCT showed testicular carcinoma in situ (CiS) in 42% of the patients (Daugaard et al, 1992). According to those authors, testicular CiS was not found in any of the eight patients with mediastinal EGGCT. Based on these findings, the hypothesis was put forward that some cases of retroperitoneal EGGCT may be due to a primary testicular cancer with metastases to the retroperitoneal lymph nodes and subsequent necrosis of the primary tumour, thus finally presenting as EGGCT.

Testicular cancer has become a model of a curable metastatic solid malignant tumour. In most reports, the survival rates of patients with EGGCT are, however, inferior compared with those of patients with advanced testicular cancer, although the treatment regimens are often the same for both conditions.

In this report, we describe the findings at the time of diagnosis and the outcome of all 48 patients diagnosed and treated for EGGCT at the Norwegian Radium Hospital (NRH) during the period 1979–95, i.e. from the time when cisplatin-based chemotherapy became available in Norway.

### PATIENTS AND METHODS

### Patients

This series comprises all 48 patients treated for EGGCT at the Norwegian Radium Hospital (NRH) between 1979 and 1994. Thirty-six patients treated within 1991 had previously been entered into the series of the International Germ Cell Cancer Collaborative Group (IGCCCG) (International Germ Cell Cancer Collaborative Group, 1997). Forty-three cases were classified as extragonadal germ cell tumours by malignant germ cell histology of a mid-line tumour. In five patients with mid-line tumours,

*Revised 3 July 1997*
*Accepted 9 July 1997*

*Correspondence to:* SD Fosså, The Norwegian Radium Hospital, Montebello, 0310 Oslo, Norway

**Table 1** Definition of the Germ Cell Consensus Classification

| | Prognosis | | |
|---|---|---|---|
| | **Good** | **Intermediate** | **Poor** |
| Non-Seminoma | with all of:<br>• Testis/retroperitoneal primary<br>• No non-pulmonary visceral metastases<br>• AFP < 1000 ng $ml^{-1}$ and<br>HCG < 1000 ng $ml^{-1}$ and<br>LDH < 1.5 × upper limit of normal<br>56% of non-seminomas<br>5-year PFS 89%<br>5-year survival 92% | with all of:<br>• Testis/retroperitoneal primary<br>• No non-pulmonary visceral metastases<br>• AFP ≥ 1000 and ≤ 10 000 IU/l or<br>HCG ≥ 5000 IU $l^{-1}$ and ≤ 50 000 IU $^{-1}$l or<br>LDH ≥ 1.5 × N and ≤ N and 10 × N<br>28% of non-seminomas<br>5-year PFS 75%<br>5-year survival 80% | with ANY of:<br>• Mediastinal primary<br>• Non-pulmonary visceral metastases<br>• AFP > 10 000 ng $1^{-1}$ or<br>HCG > 50 000 IU $l^{-1}$(100 000 ng $ml^{-1}$ or<br>LDH > 10 × upper limit of normal<br>16% of non-seminomas<br>5-year PFS 41%<br>5-year survival 48% |
| Seminoma | with all of:<br>• Any primary site and<br>• No non-pulmonary visceral metastases<br>• and Normal AFP<br>Any HCG<br>Any LDH<br>90% of seminomas<br>5-year PFS 82%<br>5-year survival 86% | with all of:<br>• Any primary site and<br>• No non-pulmonary visceral metastases<br>• and Normal AFP<br>Any HCG<br>Any LDH<br>10% of seminomas<br>5-year PFS 68%<br>5-year survival 73% | with ANY of:<br>No patients classified as poor prognosis |

PFS, progression-free survival; N, normal.

histology revealed necrosis only, and the diagnosis of EGGCT was made by elevated serum levels of human choriogonadotropin (HCG) or alfafetoprotein (AFP). In all cases, the testicles or ovaries were considered to be tumour free by clinical or ultrasonographic examination. Testicular biopsies before treatment were performed in the 36 patients treated after 1985 without evidence of invasive testicular malignancy. (The results will be further discussed in a separate paper.) All patients had computerized tomography (CT) of the chest and the abdomen before start of treatment. Based on the largest tumour manifestation, the primary site of the malignancy was categorized as either abdominal or mediastinal. If possible, histology was described according to the World Health Organization (WHO) classification for malignant germ cell tumours. However, in nine patients with proven germ cell malignancy, the subtype could not be determined. In these nine cases of proven germ cell malignancy and in the five abovementioned patients with pure necrosis in the biopsy, elevated serum AFP (any level above the normal range) or elevated serum HCG > 30 000 U $1^{-1}$ (one case) was considered to prove the presence of a non-seminoma. Following these definitions, 3 of the above 14 cases were classified as seminomas and 11 as non-seminomas. The upper normal reference values were 10 U $l^{-1}$ for HCG; 20 μg $l^{-1}$ for AFP and 450 U $l^{-1}$ for LDH.

Based on histology, localization of the metastases and the initial level of serum AFP, HCG and LDH, all 48 patients were categorized into prognostic groups according to the classification of the IGCCCG (Table 1), with the modification that the two women were included.

### Treatment

One patient with abdominal seminoma without mediastinal involvement and normal serum HCG and AFP values received abdominal and mediastinal radiotherapy (40 Gy) as his only treatment. Depending on the institution's policy at the time of diagnosis, the other 47 patients received cisplatin-based chemotherapy as described previously for testicular cancer (Fosså et al, 1988; Lewis et al, 1991). The type of primary treatment and the number of cycles of chemotherapy given are summarized in Table 2. Because of the advanced stage in many of these patients with EGGCT, rather intensive chemotherapy schedules were frequently selected. In general, the application of at least four cycles were planned in each patient. If after four or more chemotherapy cycles the serum tumour markers were normal or had reached a plateau, but residual masses were detectable in the absence of new lesions, post-chemotherapy resection of these masses was principally scheduled in patients with non-seminoma, but not in those with seminoma.

### Response

At the end of the initial treatment containing at least four chemotherapy cycles, three response categories were identified.

### *Complete response*

Complete response required the normalization of clinical, radiological and serological findings by chemotherapy alone or after post-chemotherapy resection of residual masses. Histology of such masses could either be fibrosis/necrosis, mature or viable malignant tumour. (There was no patient with residual viable malignant tumour with only a biopsy or an incomplete resection.)

### *Inevaluable for response*

A patient was inevaluable for response when there was the persistence of unbiopsied and unresectable tumour masses in the presence of normal tumour markers.

© Cancer Research Campaign 1998

**Table 2** Type of treatment and number of cycles of chemotherapy given

| | Abdominal | Mediastinal |
|---|---|---|
| *Type of primary treatment* | | |
| CVB | 4 | 1 |
| BEP 20 | 10 | 3 |
| High-dose cisplatin | 10 | 3 |
| BOP/VIP | 4 | 7 |
| Others | 3 | 1 |
| CEB | 1 | |
| Radiotherapy | 1 | |
| *No. of chemotherapy cycles* | | |
| 0 | 1[a] | 1[b] |
| 1 | 3 | |
| 2 | 1 | |
| 4 | 10 | 3 |
| 5 | 5 | 1 |
| 6 | 8 | 8 |
| 7 or 8 | 5 | 2 |

CVB, cisplatin (100 mg $m^{-2}$ per cycle), vinblastine, bleomycin; BEP20, as CVB, but vinblastine substituted by etoposide; high-dose cisplatin, as CVB or BEP20, but cisplatin 180–200 mg $m^{-2}$ per cycle; BOP/VIP, bleomycin, oncovin, cisplatin/etoposide, ifosphamide, cisplatin; CEB, as BEP20, but cisplatin substituted by carboplatin. [a]Radiotherapy only. [b]High-dose cisplatin-based chemotherapy planned but not given because of treatment-related respiratory failure starting during the prehydration phase.

### *Progression*

Progression was defined as rising serum tumour markers or the development of new tumour manifestations. (Plateau development of serum tumour markers was not observed in any of the patients.) Patients receiving less than four of the planned cycles of chemotherapy because of treatment toxicity were considered to be non-assessable for response.

### Survival

All patients were followed-up to death or to 1 January 1997, the median observation time for surviving patients being 96 months (range 29–192 months). Overall survival and cancer-specific survival were assessed, the latter evaluating death due to the malignant disease only (with the exclusion of patients with a toxic death due to post-operative or chemotherapy-related complications).

### Statistics

Standard statistical tests were used (median, range, Wilcoxon rank-sum test, chi-square test). Survival was assessed using the Kaplan–Meier procedure with the log-rank test for evaluation of differences between survival curves. All survival rates are given with their 95% confidence interval (95% CI). A $P$-value of $\leq 0.05$ was regarded as being statistically significant.

### RESULTS

### Initial work-up

The median age of all patients was 36 years (range 19–75 years) (Table 3). The patients with seminoma had a median age of 52 years (range 24–70 years) compared with the median age of 32 years for patients with non-seminoma ($P < 0.02$). Thirty-three patients had an abdominal extragonadal tumour, in 15 patients combined with a primary mediastinal tumour. In 15 patients, the mediastinal was presumed (Table 2), nine of these also presenting with abdominal mass. Two patients were women, both with mediastinal primaries. All 13 patients with extrapulmonal haematogeneous metastases (liver, seven; bone, four; brain, one; brain + liver, 1) had a non-seminomatous tumour. Using the IGCCCG system (with the above modifications) for germ cell tumours, 12 patients belonged to the good-prognosis group, 11 patients to the intermediate-prognosis group and 25 patients to the poor-prognosis group.

### Post-chemotherapy surgery

A total of 33 patients had post-chemotherapy surgery, scheduled as a part of their primary treatment. Retroperitoneal surgery was performed in 23 patients and thoracotomy in eight. One patient underwent post-chemotherapy axillary gland dissection and in another a residual mass was resected from the left supraclavicular fossa. Necrosis or fibrosis was found in 18 patients, mature teratoma in six and viable malignant tumour tissue in nine patients; in one of these nine patients the resected initially non-seminomatous primary mediastinal tumour contained viable malignant germ cell tissue combined with rhabdomyosarcoma. The patient died during the post-operative course. At autopsy, malignant histocytosis of the lymphatic tissue, of the bone marrow and in the liver was demonstrated.

### Response

One patient with abdominal seminoma was rendered tumour free by radiotherapy alone. Twenty-seven of 42 patients receiving four or more chemotherapy cycles showed a complete response. Among these complete responders, there were five (out of 13) patients, who initially presented with metastases to the brain, liver or bones. Two further patients were recorded to be tumour free after post-chemotherapy surgical excision of viable cancer tissue. Four patients were inevaluable for response, whereas progression was recorded in nine patients. The remaining five patients were non-assessable for response as they received three or less chemotherapy cycles. One of them was, however, tumour free at autopsy, which was performed after he had died because of septicaemia after his second cycle.

### Relapse and salvage treatment

Four of the 30 completely responding patients relapsed as did all four patients who were inevaluable for response at the end of primary chemotherapy. The former four cases included both patients in whom viable malignant tumour tissue was resected. They could not be salvaged by available chemotherapy (Hollender et al, 1997). Two other patients relapsing after initial complete response became tumour free, but both developed a second relapse 7 and 9 years after this first salvage treatment. Two of the four patients inevaluable for response progressed shortly after their initial chemotherapy and were not salvaged. The third patient with the mediastinal seminoma responded completely to repeated chemotherapy and radiotherapy, but developed a second incurable relapse 3 years thereafter. In the fourth patient with inevaluable response, the residual mediastinal tumour increased in size 3 years after chemotherapy discontinuation. The subsequent resection of the tumour revealed a growing mature teratoma. He has remained without evidence of disease thereafter.

© Cancer Research Campaign 1998

**Table 3** Patient demographics at diagnosis

| | Abdominal | Mediastinal |
|---|---|---|
| Number of patients | 33 | 15 |
| Median age (range) (years) | 35 (23–75) | 35 (18–52) |
| Primary histology | | |
| Pure seminoma[a] | 8 | 1 |
| Embryonal carcinoma[a] | 6 | 1 |
| Choriocarcinoma[a] | 5 | 6 |
| Yolk sac[a] | 3 | 4 |
| Necrosis/no histology | 3 | 2 |
| Germ cell, malignancy not classifiable | 8 | 1 |
| Categorized germ cell malignancy subtype[b] | | |
| Seminoma | 11 | 1 |
| Non-seminoma | 22 | 14 |
| Largest diameter (mm) | | |
| Abdominal | 100[c] (23–250)[d] (33)[e] | 40 (23–78) (7) |
| Mediastinal | 45 (35–100) (8) | 127 (16–210) (15) |
| Pulmonary | 20 (5–70) (7) | 40 (30–220) (5) |
| Number of lung metastases | 10 (1–80) (7) | 12 (1–50) (5) |
| Extrapulmonary haematogeneous metastases | | |
| Liver | 4 | 3 |
| Bone | 2 | 2 |
| Brain | | 1 |
| Liver + brain | 1 | |
| Serum tumour-markers | | |
| HCG (IU $l^{-1}$) | 150 (5–880 000) | 74 (5–453 000) |
| AFP (μg $l^{-1}$) | 5 (5–100 000) | 1100 (5–100 000) |
| LDH (U $l^{-1}$) | 1302 (307–8525) | 892 (315–2202) |
| No. of patients with elevated HCG (> 10 U $l^{-1}$)[f] | 23 (18/5) | 9 (8/1) |
| No. of patients with elevated AFP (> 20 μg/$l^{-1}$)[f] | 7 | 8 |
| No. of patients with elevated LDH (≥ 1.5 × *n*)[f] | 26 (18/8) | 9 (8/1) |

[a]Dominant component in the pretreatment biopsy. [b]Based on primary histology or serum tumour markers. [c]Median. [d]Range. [e]No. of patients. [f]Total (non-seminoma/seminoma).

**Figure 1** Five-year overall (●) and cancer-specific (▲) survival of all 48 patients with extragonadal germ cell tumour

**Figure 2** Five-year cancer-specific survival in 48 patients with extragonadal germ cell tumour according to the IGCCCG classification. ♦, Good/ intermediate prognosis; ◇, poor prognosis

### Survival

At the end of the observation time, 27 patients were alive, and 21 patients have died. Twenty-six of the surviving patients were without evidence of disease at the last observation (abdominal primary, 19 out of 33; mediastinal primary, 7 out of 15). One patient is alive with rising serum AFP, 13 years after the initial diagnosis of an abdominal EGGCT with cerebral metastases. Fourteen patients have died of their malignancy, whereas seven patients died because of treatment-related complications (vide infra). The 5-year overall survival rate for all 48 patients is 60% (95% CI 50–100%) and 71% (95% CI 50–92%) as regards cancer-specific survival (Figure 1). One additional death due to EGGCT occurred 12 years after the primary diagnosis of a mediastinal mature teratoma. The outcome for the 12 patients with seminoma revealed a 5-year overall survival rate of 75% (95% CI 50–100%) (including the successfully irradiated patient) and a 55% (95% CI 39–71%) 5-year overall survival rate for the 36 patients with non-seminomatous histology. The 5-year overall survival for the 33 patients with abdominal tumours was 63% (95% CI 46–80%), whereas it was 53% (95% CI 28–78%) for the 15 patients with mediastinal primaries. Using the IGCCCG classification system with the above modifications, the overall 5-year survival for the combined good–intermediate group was 69% (95% CI 50–88%) compared with 52% (95% CI 32–72%) for the poor-risk patients (Figure 2), the respective figures for 5-year cancer-specific survival being 80% (95% CI 62–98%) and 62% (95% CI 41–63%) (Figure 3).

### Toxic deaths

A total of seven patients died because of treatment-related complications (Table 4). Patient no. 1 who, at the time of diagnosis, had more than 50 lung metastases and a large mediastinal tumour, died from pulmonary insufficiency during the prehydration phase of the first

 © Cancer Research Campaign 1998

**Figure 3** Five-year overall survival in 48 patients with extragonadal germ cell tumour according to the IGCCCG classification. ◆, Good/intermediate prognosis (23 patients); ◇, poor prognosis (25 patients)

scheduled chemotherapy cycle before any cytostatic drugs were given. Three patients (nos 4, 6 and 7) died after the first cycle and one (no. 2) after two cycles because of chemotherapy-induced neutropenic septicaemia. These patients received chemotherapy before granulocyte colony-stimulating factor (G-CSF) was available. The remaining two patients received four and five cycles. They underwent post-chemotherapy surgery for residual mediastinal masses, but died of post-operative complications. Two of the seven patients who died of treatment-related complications (patients no. 2 and 6) were tumour free based on the histological examination of the operation specimen or on the results of autopsy. The median age of the seven patients with fatal treatment-related complications was 52 years compared with 33 years for the remaining 41 patients ($P < 0.05$). Only one of the seven patients who died of treatment-related causes was below the age of 40 years. The ages of the four patients who died of septicaemia after receiving only 1 or 2 cycles of chemotherapy were 47, 51, 70 and 76 years.

### Subsequent testicular cancer

Three patients developed an invasive testicular cancer 3, 10 or 11 years after the diagnosis of EGGCT. In the two patients with an abdominal non-seminomatous EGGCT, the subsequent testicular histology revealed a pure seminoma in one case and non-seminoma in the other. The third patient with an initial mediastinal pure seminoma (without abdominal manifestations) developed a stage II non-seminoma 3 years after the diagnosis of EGGCT and simultaneous testicular carcinoma in situ of his left testicle. (His maldescent right testicle had been removed during puberty.)

### DISCUSSION

In this report, we describe the clinical course of 48 cases with extragonadal extracerebral germ cell tumours diagnosed over a period of 15 years in a geographically defined area ('Health region II') of about 1.5 million people. Our study comprises all patients seen at the NRH during this period, including a patient who died after only having received prehydration before any chemotherapy was administered. All patients with this diagnosis in our Health region were referred to the NRH. Our experience therefore indicates an incidence of extragonadal germ cell tumour of 0.5 per 100 000 per year. This represents about 2% of the number of testicular cancer patients in the same population during the same time period.

It has been suggested that mediastinal and retroperitoneal EGGCTs have different pathogenesis and tumour biology, the former sometimes combined with haematological malignancies at

**Table 4** Patients with toxic deaths

| Patient no. | Age (years) | Primary site of metastasis | Histology | HCG ($IU\ l^{-1}$) | AFP ($\mu g\ l^{-1}$) | Treatment | Complications | Time to death after start of treatment |
|---|---|---|---|---|---|---|---|---|
| 1 | 41 | Mediastinal Lung/liver | Non-seminoma | 181 000 | 5 | 0[a] | Acute respiratory failure | 3 days |
| 2 | 75 | Abdominal | Seminoma | 12 000 | 5 | Two cycles BEP20 | Neutropenic septicaemia | 1 month |
| 3 | 18 | Mediastinal | Non-seminoma | 66 | 2.960 | Five cycles BOP/VIP Thoracotomy | Post-operative complications | 5 months |
| 4 | 47 | Abdominal Liver | Non-seminoma | 782 000 | 5 | One cycle BEP20 | Neutropenic septicaemia | 20 days |
| 5 | 53 | Mediastinal | Non-seminoma | 31 270 | 5 | Four cycles BOP/VIP Thoracotomy | Post-operative complications | 4 months |
| 6 | 51 | Abdominal | Seminoma | 17 | 5 | One cycle High-dose cisplatin | Neutropenic septicaemia | 1 month |
| 7 | 70 | Abdominal | Seminoma | 40 | 5 | One cycle CVB | Neutropenic septicaemia | 11 days |

[a]Planned high-dose cisplatin-based chemotherapy.

© Cancer Research Campaign 1998

the time of diagnosis or after treatment (Nichols et al, 1985, 1987). This is in agreement with the observation of histocytosis in one of our patients with malignant mediastinal EGGCT. Recently, it has also been suggested that some retroperitoneal EGGCTs may represent metastases from a testicular cancer with subsequent spontaneous necrosis of the primary tumour (Daugaard et al, 1992). On the other hand, Chaganti et al (1994) suggested the same origin of all germ cell tumours based on cytogenetic studies. In our series, only about 40–50% of the tumours were confined to either mediastinum or to the abdomen, making it at times difficult to clearly determine an abdominal or mediastinal origin.

At the time of presentation, most of the patients have advanced disease, 29% of our patients presenting with extrapulmonary haematogeneous spread. Many clinical investigators consider patients with EGGCT to have a poor prognosis without taking into account the extent of disease. Although it is true that the survival of these patients is below that of patients with testicular cancer (Feun et al, 1980; Gonzalez-Vela et al, 1992), only about half of our patients with EGGCT belonged to the IGCCCG poor-prognosis group. As in the IGCCCG series, patients with a seminomatous EGGCT generally had a more favourable diagnosis than those with a non-seminomatous tumour.

The pretreatment establishment of a specific histological diagnosis may sometimes be difficult in patients with EGGCT, particularly as treatment often has to be started in an emergency situation. The determination of germ cell tumour serum markers (HCG, AFP) is critical for the diagnosis of EGGCT and subtyping. Often, high serum markers have to be taken as proof of germ cell malignancy in the case of mid-line tumours in younger patients. Using these guidelines, the ratio of seminoma to non-seminoma in our patient population was 1:3. Others have reported ratios from 1:1.1 to 1:2.5 (Gutierrez Delgado et al, 1993; Goss et al, 1994; Gerl et al, 1996). All patients with haematogeneous metastases to extrapulmonary organs had a non-seminoma, indicating the tendency of these tumours to metastasize to brain, bone or liver compared with seminoma.

Our results indicate that even patients with far-advanced extragonadal germ cell tumours can be cured by intensive chemotherapy with a 5-year overall survival of 60% and a 5-year cancer-specific survival of 71%. Of the 28 patients with a complete response to chemotherapy (27) or radiotherapy (one), 27 are alive. These figures are higher than reported by two other groups (Gonzalez-Vela et al, 1992; Gutierrez Delgado et al, 1993), but similar to results published by Goss et al (1994). In contrast to these results, none of 11 patients who had progressed at the end of primary chemotherapy treatment or had viable tumour cells in post-chemotherapy biopsies was durably salvaged by second-line treatment. This low cure rate is similar to that reported by others (Saxman et al, 1994; Gerl et al, 1996) for similar EGGCT patients.

The median age of our patients with extragonadal germ cell tumours was 35 years, which is about 10 years older than reported in the literature (Gonzalez-Vela et al, 1992; Goss et al, 1994). The high median age of our patients may explain our relatively high rate of deaths because of complications. In particular, our observations suggest that the risk of fatal neutropenic septicaemia increases with higher age. In 'older' patients, the bone marrow may be less functional than in younger patients with advanced germ cell malignancies. Special attention and care should therefore be devoted to these 'older' patients with EGGCT during chemotherapy. G-CSF should probably be administered at an early phase of their treatment to avoid complications as a result of reduced bone marrow function.

For the clinician, it is important to be aware of the possibility of late recurrences (≥ 2 years after treatment discontinuation) as seen in 3 of 32 patients at risk. Furthermore, patients with EGGCT may develop testicular tumours many years after their initial diagnosis, as also pointed out by Daugaard et al (1987) and Gerl et al (1996). These clinical observations suggest multicentricity of the germ cell malignancy, at least in some patients.

In summary, patients with extragonadal germ cell tumours often present with far advanced tumour manifestations. However, only about 50% of the consecutive patients seen at a major oncology centre belonged to the poor-prognosis group as defined by the IGCCCG. EGGCT is sensitive to cisplatin-based chemotherapy and patients should be treated with intention to cure, even when presenting with metastases to bone, liver or brain. An overall 5-year survival of 60% was obtained with a cancer-specific 5-year survival of 71%. Recurrences and new manifestations of germ cell malignancy may develop after several years. Intensive cisplatin-based chemotherapy of these patients with widespread metastases carries a considerable risk of severe treatment-related complications, in particular, of neutropenic septicaemia in patients over the age of 40 years.

### REFERENCES

Chaganti RSK, Rodriguez E and Mathew S (1994) Origin of adult male mediastinal germ-cell tumours. *Lancet* **343**: 1130–1132

Daugaard G, Von Der Maase H, Olsen J, Rorth M and Skakkebæk NE (1987) Carcinoma-in-situ testis in patients with assumed extragonadal germ-cell tumours. *Lancet* **2**: 528–529

Daugaard G, Rørth M, Von Der Maase H and Skakkebæk N (1992) Management of extragonadal germ-cell tumors and the significance of bilateral testicular biopsies. *Ann Oncol* **3**: 283–289

Dvoracek C (1949) Primary chorioepithelioma of prostate with gynecomastia. *Cas Lek Cesk* **88**: 198–202

Feun LG, Samson MK and Stephen RL (1980) Vinblastine (VLB), bleomycin (BLEO), Cis-Diamminochloroplatinum (DDP) in disseminated extragonadal germ cell tumours. *Cancer* **45**: 2543–2549

Fosså SD, Aass N and Kaalhus O (1988) Testicular cancer in young Norwegians. *J Surg Oncol* **39**: 43–63

Gerl A, Clemm C, Lamerz R and Wilmanns W (1996) Cisplatin-based chemotherapy of primary extragonadal germ cell tumors. *Cancer* **77**: 526–532

Gonzalez-Vela JL, Villalona-Calero MA, Torkelson JL, Fraley EE and Kennedy BJ (1992) Extragonadal abdominal germ cell cancers. *Am J Clin Oncol* **15**: 308–310

Goss PE, Schwertfeger L, Blackstein ME, Iscoe NA, Ginsberg RJ, Simpson WJ, Jones DP and Shepherd FA (1994) Extragonadal germ cell tumours. *Cancer* **73**: 1971–1979

Gutierrez Delgado F, Tjulandin SA and Garin AM (1993) Long term results of treatment in patients with extragonadal germ cell tumours. *Eur J Cancer* **29A**: 1002–1005

Hart WR (1975) Primary endodermal sinus (yolk sac) tumour of the liver: first reported case. *Cancer* **35**: 1453–1458

Hollender C, Stenwig AE and Ous S (1997) Survival in patients with viable malignant non-seminomatous germ cell tumour persistent after cisplatin-based induction chemotherapy. *Eur Urol* **31**: 141–147

International Germ Cell Cancer Collaborative Group (1997) International Germ Cell Consensus Classification: a prognostic factor-based staging system for metastatic germ cell cancers. *J Clin Oncol* **2**: 594–603

Johnson DE, Laneri JP and Mountain CF (1973) Extra-gonadal germ cell tumours. *Surgery* **73**: 85–90

Lewis CR, Fosså SD, Mead G, Ten Bokkel Huinink W, Harding MJ, Mill L, Paul J, Jones WG, Rodenburg CJ, Cantwell B, Keizer HJ, Van Oosterom A,

 © Cancer Research Campaign 1998

Soukop M, Slinter T and Kaye SB (1991) BOP/VIP – A new platinum-intensive chemotherapy regimen for poor prognosis germ cell tumours. *Ann Oncol* **2**: 203–211

Nichols CR, Hoffman R, Einhorn LH, Williams SD, Wheeler LA and Garnick MB (1985) Hematological malignancies associated with primary mediastinal germ cell tumors. *Ann Itern Med* **102**: 603–609

Nichols CR, Heerema NA, Palmer C, Loehrer PJ Sr, Williams SD and Einhorn LH (1987) Klinefelter's syndrome associated with mediastinal germ cell neoplasms. *J Clin Oncol* **5**: 1290–1294

Saxman SB, Nichols CR and Einhorn LH (1994) Salvage chemotherapy in patients with extragonadal nonseminomatous germ cell tumors: The Indiana University Experience. *J Clin Oncol* **12**: 1390–1393

© Cancer Research Campaign 1998